# Meetings of Societies

**Published:** 1936

**Authors:** 


### Meetings of Societies

### Bristol Medico-Chirurgical Society

The fourth meeting of the session was held on Wednesday, 8th January, at the University. The President, Mr. A. E. Iles, was in the Chair, and fifty-four members were present.

Dr. Fraser showed pathological specimens, and Dr. Wansbrough demonstrated skiagrams. Dr. Peters then read a paper on "**Colloidal changes in Blood Serum in acute infections, and their Clinical Significance**" (published on p. 17 of this issue). The paper was illustrated by lantern slides and excellent micro-cinematograph films.

In the discussion which followed the following spoke: The President, Professor Davie, Dr. Carleton and Mr. Wright.

---

The fifth meeting of the session was held on Wednesday, 12th February, in the University. The President was in the Chair, and seventy-three members were present.

Professor Davie showed pathological specimens, and Dr. Bush showed skiagrams and later made comments on them. Mr. Tudor-Thomas showed a case of successful corneal grafting.

The President then introduced Mr. Tudor-Thomas, who delivered his lecture on "**Corneal Grafting: its History, Experimental Development, and Practice.**" This lecture will be published in a subsequent issue. Mr. Walker proposed and Mr. Walbank seconded the vote of thanks which an enthusiastic audience carried with much acclamation.

---

The sixth meeting of the session was held on Wednesday, 11th March, in the University. The President was in the Chair. Dr. G. B. Bush showed skiagrams.

A discussion was opened on "Carcinoma of the Bronchus," by Dr. G. B. Bush, Professor C. B. Perry, Mr. Gordon Scarff, Dr. A. L. Taylor and Mr. Duncan Wood, whose papers we hope to publish in the next issue. It was continued by the President, Mr. E. Watson-Williams, Dr. Alexander, Professor Davie, and Dr. Taylor replied to one of the questions which had been raised.

